# Association Between Ankle-Brachial Index and Coronary Lesions Assessed by Coronary Angiography

**DOI:** 10.14740/cr376w

**Published:** 2015-02-09

**Authors:** Dinaldo Cavalcanti de Oliveira, Augusto Correia, Jose Nascimento Neto, Myrtson Gurgel, Filipe Wanick Sarinho, Edgar Guimares Victor

**Affiliations:** a Hospital of Clinics, Federal University of Pernambuco, Recife, PE, Brazil

**Keywords:** Coronary artery disease, Coronary angiography, Cardiac events, Ankle-brachial index

## Abstract

**Background:**

The ankle-brachial index (ABI) is a simple, non-invasive, and inexpensive method used in the diagnosis of peripheral arterial disease (PAD) and can identify individuals at risk for cardiovascular disease in other arteries of the body, especially the coronary and carotid arteries. The primary objective of this study was to assess whether patients with an ABI < 0.9 have more severe coronary artery disease detected on coronary angiography compared to patients with a normal ABI.

**Methods:**

This is a prospective, analytical, cross-sectional study that was performed from July 1, 2013 to June 31, 2014 that recruited 163 patients (101 men (62%) and 62 women (38%)) according to the inclusion and exclusion criteria. All patients underwent coronary angiography, and then ABI measurements were performed. Pearson’s Chi-square and Student’s *t*-tests were used to compare variables between groups. The Poisson regression model was used to evaluate whether ABI was an independent predictor of stenoses > 50%.

**Results:**

The prevalence of ABI < 0.9 was 9.8%. Patients with an ABI < 0.9 had a higher prevalence of stenoses ≥ 50% in the left anterior descendant (LAD) (68.7% vs. 36%, P = 0.02) and left main (8.7% vs. 0.6%, P < 0.001) than those with a normal ABI. On multivariate Poisson regression, an ABI < 0.9 was an independent predictor of stenosis ≥ 50% in the LAD (odds ratio (OR): 2.05 (1.39 - 3.04), P < 0.001).

**Conclusions:**

Patients with an ABI < 0.9 had a higher prevalence of stenoses ≥ 50% in the LAD and left main than those with a normal ABI. An abnormal ABI was an independent predictor of lesions ≥ 50% in LAD.

## Introduction

Atherosclerosis, a chronic disease that systemically affects the arteries, is an important cause of morbidity and mortality in humans [1, 2].

The etiological, pathophysiological, and clinical aspects of atherosclerosis are multifactorial and include genetics, aging, hypertension, diabetes mellitus, dyslipidemia, smoking, a sedentary lifestyle, immune system, inflammation and other risk factors [3-6].

Atherosclerotic changes in the arterial wall progress slowly and quietly after starting in the early stages of life. However, the clinical manifestations of the disease usually appear at the advanced stage [6, 7].

Since atherosclerosis is a systemic disease, any artery may be affected. However, the most common clinical events arise from atheroma in the coronary arteries, carotid arteries, lower limb (LL) arteries, and aorta [2, 8].

The ankle-brachial index (ABI) is a simple, non-invasive, and inexpensive method used in the diagnosis of peripheral arterial disease (PAD) and can identify individuals at risk for cardiovascular disease in other arteries of the body, especially the coronary and carotid arteries. The normal range of this index is 0.9 - 1.4 [9].

An ABI < 0.9 has 90% sensitivity and 98% specificity in detecting ≥ 50% stenosis in LL arteries [10], and is associated with an increased risk of myocardial infarction and stroke [9, 11].

The primary objective of this study was to assess whether patients with an ABI < 0.9 have more severe coronary artery disease (CAD) detected on coronary angiography compared to patients with a normal ABI. The secondary objectives were to describe and compare the clinical characteristics of patients with normal and abnormal ABI and verify whether ABI is an independent predictor of CAD.

## Material and Methods

This is a prospective, analytical, cross-sectional study that was performed from July 1, 2013 to June 31, 2014 (real world study with duration of recruitment defined by protocol) that recruited 163 patients according to the inclusion and exclusion criteria described below.

This study was performed in accordance with the ethical principles of clinical research and was approved by the clinical research ethics committee of the institution where it was performed (in accordance with the ethical standards laid down in the 1964 Declaration of Helsinki and its later amendments).

Inclusion criteria include patients with a clinical diagnosis of stable angina (Canadian Cardiovascular Society classification III/IV), age ≥ 50 years, diabetes mellitus and/or smoking associated two or more other classic risk factors for coronary disease, and moderate or severe ischemia on myocardial scintigraphy (therefore with an indication for coronary angiography).

Exclusion criteria include presence of severe lung or liver comorbidities (life expectancy < 3 years), established diagnosis or relevant clinical suspicion of cancer, prior coronary artery bypass surgery or percutaneous coronary intervention and personal antecedent of revascularization to treat PAD.

All patients admitted to the invasive cardiology and hemodynamic laboratory of our institution diagnosed with chronic CAD and clinical indication for coronary angiography were evaluated as potential participants. Patients in accordance to inclusion and exclusion criteria were invited to participate and those who agreed were provided written informed consent.

Thereafter, through application of questionnaires the variables of interest were collected, and then ABI measurements were performed. Coronary angiography reports were collected, which included the description of coronary stenoses. The interventional cardiologist was blinded to the study protocol.

For the ABI measurements, a cuff with a length and width compatible with the diameter of the studied segment was used to measure the arterial systolic pressure of the upper and LLs. The ABI was measured using a portable vascular Doppler scanning MEDPEJH (Sao Paulo, Brazil) 10 MHz DV 2001 model and a BICH aneroid sphygmomanometer (Sao Paulo, Brazil).

All measurements were performed with the patient in a supine position after a 10-min rest. The systolic pressures of the posterior tibial and dorsalis pedis arteries were measured. The highest of these pressures was divided by the highest systolic pressure found in the brachial artery of the upper limbs to obtain the ABI. The index was expressed as a function of the member with the lowest index.

The presence of stenosis ≥ 30% was the criteria used to define coronary atherosclerosis. In case there was more than one stenosis per analyzed vessel, the most severe was taken into account. To assess severity, we analyzed the largest percentage of stenosis of each artery affected by atherosclerotic disease. Stenoses were classified as 30-49% and 50-100% (named as ≥ 50%).

Patients were divided into those with an ABI < 0.9 (abnormal ABI group) and those with an ABI 0.9 - 1.4 (normal ABI). Patients with an ABI > 1.4 were not included in the statistical analysis.

Categorical variables were expressed as percentages, while normally distributed numeric variables were expressed as average and standard deviation. The patients’ clinical characteristics are shown according to their frequency distribution.

Pearson’s Chi-square and Student’s *t*-tests were used to compare variables between groups. As the rates of stenoses > 50% in the coronary arteries were different between the groups, a Poisson regression model was used to evaluate whether ABI was an independent predictor of stenoses > 50%.

Values of P ≤ 0.05 were statistically significant. Statistical Package for the Social Sciences (SPSS) v 21 was used to store and analyze the variables.

## Results

From July 1, 2013 to June 31, 2014, 656 patients underwent coronary angiography in our institution but a total of 163 patients (25%) were included in this study according to inclusion and exclusion criteria.

There were 101 men (62%) and 62 women (38%). The mean age was 62.5 ± 9.71 years. The prevalence of ABI < 0.9 was 9.8%.

The average age of patients with abnormal ABI was higher than those with this index normal (68.9 ± 9.4 versus 61.8 ± 9.5 years, P = 0.005). Table 1 shows ABI according to different age groups.

**Table 1:** Prevalence of Abnormal and Normal ABI According to Different Age Groups

Age groups	ABI ≥ 0.9	ABI < 0.9	P
40 - 59 years	68 (46.2)	3 (18.7)	0.06
60 - 69 years	50 (34)	5 (31.2)	0.9
≥ 70 years	29 (19.8)	8 (50.1)	0.01

ABI: ankle-brachial index.

The patients’ clinical characteristics were: hypertension 134 patients (82.2%), smoking 89 (54.6%), diabetes mellitus 74 (45.4%), family history of CAD 72 (44.1%), dyslipidemia 69 (42.3%), obesity 39 (23.9%), arrhythmia 16 (9.8%), peripheral vascular insufficiency 11 (6.7%), stroke 10 (6.1%), chronic kidney disease six (3.7%), liver disease six (3.7%) and chronic obstructive pulmonary disease five (3.1%). There were no differences of the clinical profiles between patients with normal and abnormal ABI (Table 2).

**Table 2:** Comparison of the Main Clinical Characteristics Between Patients With Normal and Abnormal ABI

Variables	ABI ≥ 0.9	ABI < 0.9	P
Hypertension, n (%)	119 (80.9)	15 (93.7)	0.3
Smoking, n (%)	80 (54.4)	9 (56.5)	0.9
Diabetes mellitus, n (%)	68 (46.2)	6 (37.5)	0.6
FH CAD, n (%)	64 (43.2)	8 (50)	0.8
Dyslipidemia, n (%)	63 (42.5)	6 (37.5)	0.8
Obesity, n (%)	34 (23.1)	5 (31.2)	0.6
Stroke, n (%)	9 (6.1)	1 (6.2)	0.5
VPI, n (%)	9 (6.1)	2 (12.5)	0.6
BMI, kg/m^2^	28.1 ± 4.8	28.3 ± 5.2	0.9

ABI: ankle-brachial index; FH CAD: family history of coronary artery disease; MI: myocardial infarction; VPI: peripheral vascular insufficiency; BMI: body mass index.

Results of coronary angiographies have shown that in the left coronary artery, stenoses ≥ 30% occurred in 7.9% patients (30-49% in 5.5% and ≥ 50% in 2.4%). In the left anterior descending artery (LAD), stenoses were ≥ 30% in 57.6% of patients (30-49% in 18.4% and ≥ 50% in 39.2%). In the circumflex artery (LCX), there were stenoses ≥ 30% in 38% of patients (30-49% in 14.7% and ≥ 50% in 23.3%). In the right coronary artery (RCA), stenoses ≥ 30% occurred in 47.2% of patients (30-49% in 12.8% and ≥ 50% in 34.4%).

A comparative analysis of coronary stenoses (30-49% and > 50%) between the groups is shown in Table 3.

**Table 3:** Prevalence of Stenoses According to ABI

Coronaries	ABI ≥ 0.9	ABI < 0.9	P value
LM	≥ 50%, one pt (0.6%)	≥ 50%, three pts (8.7%)	< 0.001
	30-49%, nine pts (6.2%)	≥ 50%, 0 pt	0.6
LAD	≥ 50%, 53 pts (36%)	≥ 50%, 11 pts (68.7%)	0.02
	30-49%, 26 pts (17.6%)	30-49%, four pts (25%)	0.7
LCX	≥ 50%, 32 pts (21.7%)	≥ 50%, six pts (37.5%)	0.2
	30-49%, 19 pts (12.9%)	30-49%, five pts (31.2%)	0.1
RCA	≥ 50%, 47 pts (31.9%)	≥ 50%, nine pts (56.2%)	0.09
	30-49%, 19 pts (12.9%)	30-49%, two pts (12.5%)	0.8

LM: left main; LAD: left anterior descendant; LCX: left circumflex; RCA: right coronary artery; ABI: ankle-brachial index; pts: patients.

On multivariate Poisson regression, an ABI < 0.9 was only an independent predictor of stenosis ≥ 50% in the LAD (odds ratio (OR): 2.05 (1.39 - 3.04), P < 0.001).

## Discussion

Patients with an ABI < 0.9 had a higher prevalence of stenoses ≥ 50% in the LAD and left main than those with a normal ABI. An abnormal ABI was an independent predictor of lesions ≥ 50% in LAD.

We observe that the prevalences of stenoses ≥ 50% in the RCA and in the LCX were numerically higher in patients with abnormal ABI, but without statistical differences (there was a trend in the RCA). We believe that a study with more patients than our study may find significant differences in these arteries.

In this study, the prevalence of abnormal ABI was higher in older patients, a finding that is consistent with that of previous studies, emphasizing that aging is associated with a higher probability of abnormal ABI [12].

The association between smoking and the risk of PAD as well as dyslipidemia and CAD has been established in the literature, but in our study there was no difference in clinical profile between the groups [13].

In patients at high risk for CAD, the prevalence of an ABI < 0.9 is higher than that in the general population, reaching 42% [13-15]. Despite the fact that our study population is at high risk for CAD, we did not confirm the previously published findings.

The ABI indicates atherosclerotic involvement of the LL arteries, and values < 0.9 are associated with a significantly increased cardiovascular risk, particularly myocardial infarction and stroke, independent of other risk factors [16, 17].

A study that evaluated almost 4,393 patients demonstrated that patients with an ABI < 0.9 had a 3.7-fold higher risk of cardiovascular death compared with those with normal values [18].

Data from the Framingham Offspring Study (3,113 patients) revealed that the prevalence of CAD in patients with an ABI < 0.9 was three times higher than that in patients with a normal index (30% versus 10%, P < 0.0001) [19].

Studies have shown that patients with an ABI < 0.9 had a higher prevalence of multi-vessel CAD than those with a normal index [20, 21].

Zuo et al and Papamichael et al evaluated associations between ABI and CAD extent and severity using the Gensini score and revealed an association between abnormal ABI and higher score [22, 23].

Nonetheless, the Gensini score does not evaluate severity of CAD according to specific coronary artery [22, 23]. In our study, we analyzed stenoses according to its distribution in specific coronary arteries because the patient’s prognosis also depends on which artery is compromised. For example, lesions in the LAD determine high risk for patients.

Banerjee et al demonstrated that in patients with chronic stable CAD, an abnormal ABI confers an increased risk of cardiovascular events, independent from traditional risk factors [24]. These results confirmed previous study published by Lee et al [25].

In stable coronary heart disease, cardiovascular events result from demand ischemia or occlusion of the coronary artery due to atheroma growth, which could lead to total vessel obstruction [26].

Our findings of increased rates of stenosis ≥ 50% in the LAD in patients with an ABI < 0.9 may help explain the occurrence of coronary events in patients with an abnormal ABI reported in the literature, and generated the hypothesis that this higher prevalence of these stenoses may be the underlying pathophysiology of the clinical manifestations.

In conclusion, patients with an ABI < 0.9 had more lesions ≥ 50% in the LAD coronary artery than those with a normal ABI. Besides, this abnormal index was an independent predictor of these lesions. Hence, as hypothesis, this higher frequency of stenosis ≥ 50% may contribute to occurrence of coronary events in this group of patients.

## References

[1] Hansson GK, Libby P, Schonbeck U, Yan ZQ (2002). Innate and adaptive immunity in the pathogenesis of atherosclerosis. Circ Res.

[2] Roger VL, Go AS, Lloyd-Jones DM, Benjamin EJ, Berry JD, Borden WB, Bravata DM (2012). Heart disease and stroke statistics--2012 update: a report from the American Heart Association. Circulation.

[3] Wyss CA, Neidhart M, Altwegg L, Spanaus KS, Yonekawa K, Wischnewsky MB, Corti R (2010). Cellular actors, Toll-like receptors, and local cytokine profile in acute coronary syndromes. Eur Heart J.

[4] Bentzon JF, Otsuka F, Virmani R, Falk E (2014). Mechanisms of plaque formation and rupture. Circ Res.

[5] Libby P, Tabas I, Fredman G, Fisher EA (2014). Inflammation and its resolution as determinants of acute coronary syndromes. Circ Res.

[6] Geng YJ, Libby P (2002). Progression of atheroma: a struggle between death and procreation. Arterioscler Thromb Vasc Biol.

[7] Lane HA, Smith JC, Davies JS (2006). Noninvasive assessment of preclinical atherosclerosis. Vasc Health Risk Manag.

[8] Najjar SS, Scuteri A, Lakatta EG (2005). Arterial aging: is it an immutable cardiovascular risk factor?. Hypertension.

[9] McDermott MM, Liu K, Criqui MH, Ruth K, Goff D, Saad MF, Wu C (2005). Ankle-brachial index and subclinical cardiac and carotid disease: the multi-ethnic study of atherosclerosis. Am J Epidemiol.

[10] Doobay AV, Anand SS (2005). Sensitivity and specificity of the ankle-brachial index to predict future cardiovascular outcomes: a systematic review. Arterioscler Thromb Vasc Biol.

[11] Belch JJ, Topol EJ, Agnelli G, Bertrand M, Califf RM, Clement DL, Creager MA (2003). Critical issues in peripheral arterial disease detection and management: a call to action. Arch Intern Med.

[12] Selvin E, Erlinger TP (2004). Prevalence of and risk factors for peripheral arterial disease in the United States: results from the National Health and Nutrition Examination Survey, 1999-2000. Circulation.

[13] Poredos P, Jug B (2007). The prevalence of peripheral arterial disease in high risk subjects and coronary or cerebrovascular patients. Angiology.

[14] Nunez D, Morillas P, Quiles J, Cordero A, Guindo J, Soria F, Mazon P (2010). Usefulness of an abnormal ankle-brachial index for detecting multivessel coronary disease in patients with acute coronary syndrome. Rev Esp Cardiol.

[15] Sukhija R, Aronow WS, Yalamanchili K, Peterson SJ, Frishman WH, Babu S (2005). Association of ankle-brachial index with severity of angiographic coronary artery disease in patients with peripheral arterial disease and coronary artery disease. Cardiology.

[16] Cui R, Yamagishi K, Imano H, Ohira T, Tanigawa T, Hitsumoto S, Kiyama M (2014). Relationship between the ankle-brachial index and the risk of coronary heart disease and stroke: the circulatory risk in communities study. J Atheroscler Thromb.

[17] Kojima I, Ninomiya T, Hata J, Fukuhara M, Hirakawa Y, Mukai N, Yoshida D (2014). A low ankle brachial index is associated with an increased risk of cardiovascular disease: the Hisayama study. J Atheroscler Thromb.

[18] Resnick HE, Lindsay RS, McDermott MM, Devereux RB, Jones KL, Fabsitz RR, Howard BV (2004). Relationship of high and low ankle brachial index to all-cause and cardiovascular disease mortality: the Strong Heart Study. Circulation.

[19] Murabito JM, Evans JC, Nieto K, Larson MG, Levy D, Wilson PW (2002). Prevalence and clinical correlates of peripheral arterial disease in the Framingham Offspring Study. Am Heart J.

[20] Papa ED, Helber I, Ehrlichmann MR, Alves CM, Makdisse M, Matos LN, Borges JL (2013). Ankle-brachial index as a predictor of coronary disease events in elderly patients submitted to coronary angiography. Clinics (Sao Paulo).

[21] Otah KE, Madan A, Otah E, Badero O, Clark LT, Salifu MO (2004). Usefulness of an abnormal ankle-brachial index to predict presence of coronary artery disease in African-Americans. Am J Cardiol.

[22] Zuo G, Zhang M, Jia X, Zheng L, Li Y, Zhao H, Wang C (2014). Correlation between brachial-ankle pulse wave velocity, carotid artery intima-media thickness, ankle-brachial index, and the severity of coronary lesions. Cell Biochem Biophys.

[23] Papamichael CM, Lekakis JP, Stamatelopoulos KS, Papaioannou TG, Alevizaki MK, Cimponeriu AT, Kanakakis JE (2000). Ankle-brachial index as a predictor of the extent of coronary atherosclerosis and cardiovascular events in patients with coronary artery disease. Am J Cardiol.

[24] Banerjee S, Vinas A, Mohammad A, Hadidi O, Thomas R, Sarode K, Banerjee A (2014). Significance of an abnormal ankle-brachial index in patients with established coronary artery disease with and without associated diabetes mellitus. Am J Cardiol.

[25] Lee AJ, Price JF, Russell MJ, Smith FB, van Wijk MC, Fowkes FG (2004). Improved prediction of fatal myocardial infarction using the ankle brachial index in addition to conventional risk factors: the Edinburgh Artery Study. Circulation.

[26] Fihn SD, Gardin JM, Abrams J, Berra K, Blankenship JC, Dallas AP, Douglas PS (2012). 2012 ACCF/AHA/ACP/AATS/PCNA/SCAI/STS guideline for the diagnosis and management of patients with stable ischemic heart disease: a report of the American College of Cardiology Foundation/American Heart Association task force on practice guidelines, and the American College of Physicians, American Association for Thoracic Surgery, Preventive Cardiovascular Nurses Association, Society for Cardiovascular Angiography and Interventions, and Society of Thoracic Surgeons. Circulation.

